# Prevalence of symptoms in female Fabry disease patients: a case-control survey

**DOI:** 10.1007/s10545-011-9447-9

**Published:** 2012-03-20

**Authors:** Machtelt G. Bouwman, Saskia M. Rombach, Erica Schenk, Annelies Sweeb, Frits A. Wijburg, Carla E. M. Hollak, Gabor E. Linthorst

**Affiliations:** 1 Department of Paediatrics, Academic Medical Center, Amsterdam, The Netherlands; 2 Department of Endocrinology and Metabolism, Academic Medical Center and ‘Sphinx’ the Amsterdam Lysosome Center, Amsterdam, The Netherlands; 3 Dutch Fabry Patient Organisation, FSIGN, Amsterdam, The Netherlands; 4 Academic Medical Center, Emma Childrens Hospital, Meibergdreef 9, 1105 AZ Amsterdam, The Netherlands

## Abstract

**Background:**

Fabry disease (FD) is an X-linked lysosomal storage disorder, caused by a deficiency of α-galactosidase A. Several studies demonstrated that heterozygotes have symptoms such as acroparesthesia, abdominal pain and chronic fatigue. However, as these symptoms are aspecific and relatively common in the general population, it is important to compare the prevalence of these symptoms with an appropriate control group. The aim of this study was to explore the prevalence of signs and symptoms in FD females in comparison to a control group.

**Methods:**

FD females and age-matched controls were approached to complete a questionnaire. This questionnaire was developed by the Dutch Fabry patient organisation (Fabry Support en Informatie Groep Nederland, FSIGN) with input from Fabry expert-physicians from the AMC. We compared the prevalence symptoms using Pearson’s chi-square test. Bonferroni correction was used to correct for multiple comparisons.

**Results:**

A total of 63 heterozygotes and 52 controls completed the questionnaire. Many symptoms were also common in controls. Yet, fatigue, palpitations, pains in hands and feet, joint pain, dizziness, loss of libido and proteinuria during pregnancy were more common in Fabry females (all p < 0.001).

**Conclusion:**

In addition to acroparesthesia - fatigue, palpitations, dizziness, proteinuria during pregnancy, libido loss and joint pain are more prevalent in FD females as compared to a control group. Although, these symptoms are present in a significant proportion of normal controls they deserve further attention by treating physicians to better understand their significance, treatment and relationship with FD.

## Introduction

Fabry disease (FD) is a lysosomal storage disorder, caused by a deficiency of the lysosomal enzyme alpha-galactosidase A (Desnick et al. 2001). Accumulation of globotriaosylceramide in vascular cells leads to multiple organ disease, characterised by acroparesthesia, angiokeratoma, progressive renal failure, cardiac complications and stroke. Unlike most lysosomal storage disorders, the inheritance of FD is X-linked and therefore in general heterozygotes have a mosaic state.

Following the availability of enzyme replacement therapy (ERT) in 2001, a number of Fabry cohort studies and post-marketing registry studies have been reported in the literature, describing the natural history of FD in males (Germain et al. 2003; Macdermot et al. 2001a, b; Schiffmann 2001), females (Deegan et al. 2006; Guffon 2003; Gupta et al. 2005; Kobayashi et al. 2008; Macdermot et al. 2001a, b; Wang et al. 2007; Whybra et al. 2001; Wilcox et al. 2008) or both (Galanos et al. 2002; Mehta et al. 2004; Schiffmann et al. 2009; Vedder et al. 2007). These studies have shown that despite residual enzyme activity, FD females do develop signs and symptoms of FD, such as left ventricular hypertrophy, proteinuria, stroke and, to a lesser extent, renal failure. The prevalence of acroparesthesia, a well known symptom of Fabry disease, ranges from 23 to 90% of the patients in the different cohort studies (see Table 5). These studies also reported a high prevalence of other non-specific complaints such as fatigue, palpitations, chest pain, abdominal pain and diarrhoea in FD females (see Table 5). As all of these symptoms are non-specific, care should be taken to attribute them directly to FD, especially as these studies lack control groups. At the outpatient clinic, Fabry females sometimes report symptoms or complaints of which both patient and the treating physician are unsure whether that complaint is part of the clinical spectrum of FD. The only way to appreciate whether certain complaints, aspecific or not, are part of the spectrum of FD is to study their prevalence in comparison with an appropriate control group.

In order to study the spectrum and prevalence of FD related symptoms in FD females, the Dutch Fabry patient organisation (Fabry Support en Informatie Groep Nederland, FSIGN), in collaboration with the lysosomal center ‘Sphinx’ of the AMC, initiated a questionnaire study on symptoms in FD females and an age matched control group. Based on personal experience of a group of FD females certain symptoms were added to the questionnaire. The purpose of this study was to explore the prevalence of symptoms in FD females compared to controls by use of a questionnaire.

## Methods

### Participants and recruitment

Participants for the study were recruited from the Academic Medical Centre, the national referral center for patients with Fabry disease in the Netherlands. We asked all FD females, aged 12 years and older, diagnosed by mutation analysis (n = 81) to participate. We sent a letter explaining the aim of the study including two questionnaires.

FD females were asked to fill out one questionnaire (marked “P”) and to give one questionnaire to a non-Fabry female friend, neighbour or relative (marked “C”), with approximately the same age. All questionnaires were analysed anonymously. This study was judged by the Ethical Committee of the AMC, Amsterdam as being a non-interventional study, which does not require formal approval under Dutch law and is considered to cause no harm to the study subjects.

### Questionnaire

The questionnaire was developed by two members (ES and AS) of the Dutch Fabry patient organisation (FSIGN) in close collaboration with Fabry expert-physicians (SMR and CEH) from the AMC. During a meeting organised and attended by FD females only (n = 20), the content of the questionnaire was discussed. Based on personal experience of the attendees, additional symptoms were added that were so far not considered to be part of Fabry symptomatology (see Results, Table 4).

The questionnaire contained general questions on health, sports, smoking, employment and sleeping difficulty (12 questions). Furthermore women were asked on the presence of cardiopulmonary, gastrointestinal, musculoskeletal, neuropsychological, urogenital symptoms and pregnancy outcome (54 questions). Participants were asked to respond yes/no to the presence of these symptoms during the last three months.

### Statistical analysis

All statistical analyses were performed using SPSS version 16.0. Pearson’s chi-square test was used to compare the frequency of symptoms in FD females with the frequency in control females. The Fisher's exact test was used when one or more of the cells in the 2 × 2 contingency table had an expected frequency of five or less.

Multiple testing may result in false positive results simply due to chance. Therefore, the significance level was adjusted in accordance with the Bonferroni correction (Bland and Altman 1995). To determine the significance level, a p-value of 0.05 was divided by the number of comparisons done (n = 66; p = 0.0008) (Bland and Altman 1995) and was subsequently set at p < 0.001. In addition, odds ratios were calculated, but here we used a 95% confidence interval.

## Results

A total of 62 FD females completed the questionnaire (response rate of 77%). In addition 53 female controls participated in the study. The mean age of the FD females was 39 years versus 42 years in controls (p = 0.67; Table 1). Pearson’s chi-square test was used to compare the frequency of symptoms in FD females with the frequency in control females. The results of these analyses are mentioned in Tables 1, 2 and 3. There was no difference in employment status, smoking, alcohol use and sports participation (Table 1).

**Table 1:** Characteristics of FD females and controls

	Fabry	Control	OR (95% CI)	p value^a^
Age	39.3 (±17.5)	41.7 (±16.7)	NA	0.67^b^
Employed	29/52 (56%)	31/44 (71%)	0.53 (0.23-1.23)	0.14
Doing sports	31/62 (50%)	36/53 (68%)	0.47 (0.22-1.01)	0.052
Smoking	8/62 (13%)	10/52 (19%)	0.62 (0.23-1.71)	0.36
Drinking alcohol	30/61 (49%)	33/52 (64%)	0.58 (0.26-1.19)	0.13
Not sleeping well *	32/61 (53%)	14/53 (26%)	3.07 (1.39-6.78)	0.005
Not well rested when awake *	37/60 (62%)	16/52 (33%)	3.62 (1.65-7.94)	0.002
**Perceived health status as not good**	26/61 (42%)	3/52 (8%)	12.1 (3.40-43.3)	≤0.001
**Medication**	40/62 (65%)	16/52 (31%)	4.09 (1.86-8.98)	≤0.001
Paramedic treatment *	16/62 (26%)	7/53 (13%)	2.29 (0.86-6.08)	0.09
**In treatment specialist / general practitioner**	31/60 (52%)	10/53 (19%)	4.60 (1.96-10.8)	<0.001
Health problems *	40/60 (67%)	18/51 (35%)	3.67 (1.67-8.05)	0.001

Data are number (%). OR = odds ratio. * p < 0.05. Bold: significant after Bonferroni correction.

NA: not applicable. ^a^ according to Pearson chi-square test, ^b^ according to t-test.

**Table 2:** Prevalence of symptoms in FD females and controls

	Fabry	Control	OR (95% CI)	p value^a^
Cardiopulmonary symptoms				
**Palpitations**	29/61 (48%)	7/53 (13%)	5.96 (2.32-15.3)	≤0.001
Skipped heartbeat *	20/60 (33%)	6/53 (11%)	3.92 (1.43-10.7)	0.006
Chest pain *	18/62 (29% )	5/51 (10%)	3.76 (1.29-11.0)	0.01
Shortness of breath *	30/62 (48%)	10/53 (19%)	4.03 (1.72-9.43)	0.01
Gastrointestinal symptoms				
Regurgitation	23/61 (38% )	16/52 (31%)	1.36 (0.62-2.98)	0.44
Reflux	18/62 (29%)	11/52 (21%)	1.52 (0.64-3.61)	0.34
Nausea *	27/62 (44%)	11/53 (21%)	2.95 (1.28-6.77)	0.01
Swallowing difficulties *	19/61 (31%)	6/53 (11%)	3.54 (1.29-9.71)	0.01
Hiccup	25/58 (43%)	18/51 (35%)	1.39 (0.64-3.01)	0.40
Abdominal pain *	37/62 (60%)	16/53 (30%)	3.42 (1.58-7.43)	0.002
Diarrhoea *	25/61 (41%)	10/53 (19%)	2.99 (1.27-7.03)	0.01
Constipation *	21/61 (34%)	5/53 (9%)	5.04 (1.74-14.6)	0.002
Flatulence	34/62 (55%)	25/53 (47%)	1.36 (0.65-2.84)	0.41
Musculoskeletal symptoms				
Myalgia *	35/62 (57%)	19/53 (36 %)	2.32 (1.09-4.93)	0.03
Muscle cramps *	25/62 (40%)	8/53 (15%)	3.80 (1.53-9.42)	0.003
Back ache *	38/61 (62%)	17/53 (32%)	3.50 (1.61-7.60)	0.001
**Joint pain**	36/62 (58%)	13/52 (25%)	4.15 (1.86-9.29)	≤0.001
**Pain in hands**	36/62 (58%)	6/53 11%)	10.85 (4.04-29.1)	≤0.001
**Pain in feet**	24/61 (39%)	5/53 (9%)	6.23 (2.17-17.9)	≤0.001
Neuropsychological symptoms				
Loss of strength *	28/62 (45%)	8/53 (15%)	4.63 (1.88-11.4)	0.001
Numbness	8/61 (13%)	8/53 (15%)	0.85 (0.30-2.44)	0.56
**Fatique**	55/62 (89%)	30/53 (57%)	6.02 (2.32-15.7)	≤0.001
**Dizziness**	37/62 (60%)	10/53 (19%)	6.36 (2.71-15.0)	≤0.001
Headache	36/62 (60 %)	24/53 (45%)	1.67 (0.80-3.59)	0.12
Depressed mood	34/62 (55%)	20/51 (39%)	1.88 (0.89-3.99)	0.19
Urogenital symptoms				
Urinary incontinence	16/62 (26%)	12/53 (23%)	1.19 (0.50-2.80)	0.69
Dysuria#	3/62 (4.8%)	1/53 (1.9)	2.64 (0.27-26.2)	0.62
Premenstrual symptoms	26/40 (65%)	20/34 (29%)	1.30 (0.51-3.34)	0.59
Menstrual symptoms	24/35 (67%)	23/31(74%)	0.76 (0.26-2.22)	0.42
Menarche at 12-15 years	44/62 (71%)	36/53 (68%)	NA	0.72
Fertility problems#	5/40 (13%)	1/39 (2.6%)	5.43 (0.60-48.8)	0.20
**Loss of libido**	27/45 (60%)	9/31 (23%)	3.67 (1.38-9.75)	<0.001
Dermatological symptoms				
Dry skin*	42/61 (68%)	25/53 (47%)	2.48 (1.15-5.32)	0.03
Acne	10/61 (16%)	6/53 (11%)	1.54 (0.52-4.55)	0.44
Eczema	11/62 (18%)	7/53 (13%)	1.42 (0.51-3.96)	0.50
Vitiligo	6/62 (10%)	5/53 (9%)	1.03 (0.21-3.58)	0.97
Urticaria#	3/61 (4.9%)	2/52 (3.8%)	1.29 (0.21-8.05)	1.00
Other symptoms				
Hypohidrosis*	17/59 (29%)	5/51 (10%)	3.72 (1.26-11.0)	0.01
Spontaneous tear in molar	13/60 (22%)	10/49 (20%)	1.08 (0.43-2.73)	0.87
Allergy	10/62 (16%)	9/53 (17%)	0.94 (0.35-2.52)	0.90
Snoring	20/62 (32%)	31/53 (56%)	0.34 (0.16-0.73)	0.30
Gum disease*	16/62 (26%)	6/53 (11%)	2.72 (0.98-7.58)	0.05

Data are number (%). OR = odds ratio. Bold: significant after Bonferroni correction. * p < 0.05.

^a^according to Pearson chi-square test. # Fisher’s exact test because of small sample size.

**Table 3:** Pregnancy and pregnancy outcome in FD females and controls

	Fabry (n = 62)	Control (n = 53)	OR (95% CI)	p value^a^
Pregnancy outcome				
Hypertension during pregnancy #	11/32 (34%)	5/35 (14%)	3.14 (0.95-10.4)	0.05
**Proteinuria during pregnancy**	11/32 (34%)	0/35 (0%)	NA	<0.001
Preeclampsia #	3/32 (9.4%)	0/35 (0%)	NA	0.10
Premature delivery #	6/32 (19%)	2/35 (5.7%)	3.81 (0.71-20.4)	0.14
Induced labour	7/32 (22%)	5/35 (14%)	1.68 (0.47-5.95)	0.42
Pelvic instability #	2/32 (6.2%)	4/35 (11%)	0.52 (0.09-3.03)	0.68
Microsomia #	3/32 (9.4%)	1/35 (2.9%)	3.52 (0.35-35.7)	0.34
Macrosomia #	1/32 (3.1%)	1/35 (2.9%)	1.10 (0.07-18.3)	1.00
Postpartum bleeding #	3/32 (9.4%)	5/35 (15%)	0.62 (0.14-2.84)	0.71
Retained placenta #	4/32 (13%)	6/35 (17%)	0.69 (0.18-2.71)	0.74
Miscarriage	8/32 (25%)	4/35 (11%)	2.58 (0.69-9.61)	0.15
Intrauterine death	1/32 (3.1%)	1/35 (2.9%)	1.10 (0.07-18.3)	1.00

Data are numbers (%). OR = odds ratio. Bold: significant after Bonferroni correction. NA = not applicable. ^a^According to Pearson chi-square test. # Fisher’s exact test because of small sample size.

Perceived health status was significantly decreased in FD females as 26 patients reported not feeling healthy compared to three controls (42% versus 8 % respectively; p < 0.001, OR 12.1). Significantly more patients with FD were prescribed medication, including enzyme replacement therapy (65% versus 31% respectively, p < 0.001, OR 4.09; Table 1). In total 28 FD females were treated with ERT (45%).

### Cardiopulmonary symptoms

Palpitations were reported significantly more often in FD females (48% versus 13% in controls, p < 0.001, OR 5.96). Almost half of the patients reported to have shortness of breath, although there was no significant difference after Bonferroni correction (Table 2).

### Gastrointestinal (GI) symptoms

GI symptoms were reported often by both FD females and control females, see Table 2. FD females frequently reported nausea, swallowing difficulties, abdominal pain, diarrhoea and constipation. Abdominal pain was reported by 60% of the FD females. Diarrhoea and constipation were present in 41% and 34% respectively. These symptoms were also common in controls (in 30%, 19% and 9% respectively). After adjustment of the p-value with the Bonferroni correction, there were no significant differences in gastro-intestinal symptoms between the two groups (nausea: p = 0.01; swallowing difficulties: p = 0.01; abdominal pain: p = 0.002; diarrhoea: p = 0.01 and constipation: p = 0.002). Any GI symptom was mentioned by 51 of the 62 Fabry females versus 27 of the 53 controls (82% versus 51%, p < 0.001, Pearson chi square test).

### Musculoskeletal symptoms

The results on the neuromuscular symptoms are summarised in Table 2. Joint pain was present in 36 FD females which was significantly more compared to 13 control females (58% versus 25%, p < 0.001, OR 4.15). In addition pain in hands and feet was reported significantly more frequent in FD females (58% versus 11%, p <0.001, OR 10.9 and 39% versus 9% respectively, p < 0.001, OR 6.2).

### Neuropsychological symptoms

Dizziness was reported more frequently by FD females (60% versus 19%, p < 0.001, OR 6.36). Fatigue was reported by almost all patients (n = 55), but also by many controls (n = 30), yet it was significantly more common in FD females (89% versus 57%, p < 0.001, OR 6.02). No differences were found in other neuropsychological symptoms (see Table 2).

### Menstrual cycle

The prevalence of premenstrual, menstrual symptoms and menarche was not different between Fabry females and controls (p = 0.59, p = 0.42 and p = 0.72 respectively; see Table 2).

### Pregnancy related outcome

Thirty-two females with Fabry disease had a history of pregnancy and 35 controls (in total 89 pregnancies in Fabry females, 78 in controls; miscarriages not included). The results are summarised in Table 3. Proteinuria during pregnancy was reported by nearly one third of Fabry females (34% versus 0%, p < 0.001) as well as hypertension during pregnancy in FD females, although the latter was not significantly different (34% versus 14%, p = 0.05). There were no significant differences in frequency of pre-eclampsia (9.4% versus 0%, p = 0.10), miscarriages (25% versus 11%, p = 0.15), fertility problems (13% versus 2.6%, p = 0.20) and premature delivery in FD females and controls (19% versus 6%, p = 0.14). In addition, intrauterine death did not occur more often in the studied group (3% versus 3%, p = 1.0).

### Other symptoms

Loss of libido was also reported significantly more common by FD females than controls (60% vs.23%, p <0.001, OR 3.67). Loss of libido was not significantly related to fatigue in both FD females (p = 0.38) and non-FD females (p = 0.06). Twenty-nine percent (17/59) of the FD females had hypohidrosis (versus 10% in controls 5/51, p = 0.01) (see Table 2). Table 4 shows the frequency of occurence of symptoms suggested specifically by Fabry females to be included in the questionaire.

**Table 4:** Symptoms suggested by Fabry females: FD females versus controls

	Fabry (n = 62)	Control (n = 53)	OR (95% CI)	p value^a^
Not sleeping well *	32/61 (53%)	14/53 (26%)	3.07 (1.39-6.78)	0.005
Not well rested when awake *	37/60 (62%)	16/52 (33%)	3.62 (1.65-7.94)	0.002
Snoring	20/62 (32%)	31/53 (56%)	0.34 (0.16-0.73)	0.30
Headache	36/62 (60 %)	24/53 (45%)	1.74 (0.83-3.66)	0.12
Flatulence	34/62 (55%)	25/53 (47%)	1.36 (0.65-2.84)	0.41
Hiccup	25/58 (43%)	18/51 (35%)	1.39 (0.64-3.01)	0.40
Myalgia *	35/62 (57%)	19/53 (36 %)	2.32 (1.09-4.93)	0.027
Muscle cramps *	25/62 (40%)	8/53 (15%)	3.80 (1.53-9.42)	0.003
Dry skin*	67.6 (42/61)	47.2 (25/53)	2.48 (1.15-5.32)	0.03
Acne	16.4 (10/61)	11.3 (6/53)	1.54 (0.52-4.55)	0.44
Eczema	17.7 (11/62)	13.2 (7/53)	1.42 (0.51-3.96)	0.50
Vitiligo	9.7 (6/62)	9.4 (5/53)	1.03 (0.30-3.58)	0.97
Spontaneous tear in molar	21.7 (13/60)	20.4 (10/49)	1.08 (0.43-2.73)	0.87
Allergy	16.1 (10/62)	17.0 (9/53)	0.94 (0.35-2.52)	0.90
Gum disease	25.8 (16/62)	11.3 (6/53)	2.72 (0.98-7.58)	0.05

Data are numbers (%). OR = odds ratio. * = p < 0.05. ^a^ according to Pearson chi-square test

## Discussion

In this exploratory study we compared the prevalence of both classical FD related symptoms as well as a variety of other complaints in a large cohort of FD females with prevalence of these symptoms in a well matched control group. This study confirms that some previously described symptoms are significantly more common in Fabry females. Pain in hands and feet, acroparesthesia, are indeed a common symptom in FD females. The prevalence of acroparesthesia in the current study is higher than previously reported by us in a smaller cohort (Vedder et al. 2007), and is in agreement with the overall prevalence of 61% (range 23-90%) in several other studies (see Tables 2 and 5). Palpitations were also significantly more common in FD females. Fatigue was the most commonly reported symptom (89%), as was also reported by Mac Dermot et al. (Macdermot et al. 2001a, b). Although, as may be expected, a substantial proportion of normal controls also experienced chronic fatigue (57%), this was significantly more prevalent in the FD females. In addition to the more common FD attributed symptoms, this study also confirms a higher prevalence of symptoms of joint pain and dizziness in FD females as compared to controls.

**Table 5:** Overview of prevalence of symptoms in cohort studies of FD females

Study	From	Patients *n*	AP	HH	GE symptoms	Diarrhoea	Constipation	Abdominal pain	Cardiovascular symtoms	Angina	Palpitations	Fatigue	Vertigo	Tinnitus	Depression
Macdermot et al. 2001a, b	UK	60	42/60	20/60	35/60				32/60			40/60		15/60	
			70%	33%	58%				53%			67%		25%	
Whybra et al. 2001	Germany	20	18/20		10/20		2/20								
			90%		50%		10%								
Galanos 2002	Australia	38	20/38	4/38	4/38					8/38	11/38				
			53%	11%	11%					20%	29%				
Guffon 2003	France	11	8/11		3/11								6/11		5/11
			73%		27%								55%		45%
Gupta et al. 2005	USA	57	42/57	18/57		26/57		30/57						11/57	
			74%	32%		46%		53%						19%	
Wang et al. 2007	USA	51	26/40	25/42		18/42		16/41		9/36	8/38	24/51		22/38	21/34
			65%	60%		43%		39%		25%	21%	59%		58%	62%
Vedder et al. 2007	NL	39	9/39		5/39										
			23%		12%										
Kobayashi et al. 2008	Japan	36	18/36	6/36											
			50%	17%											
Overall prevalence			183/301	73/233	57/168	44/99	2/20	46/98	32/60	17/74	19/76	64/111	6/11	34/155	26/45
			61%	31%	34%	44%	10%	47%	53%	23%	25%	58%	55%	22%	58%
This study; FD	NL	62	36/62		51/62	25/61	21/61	37/51	40/62	18/62	29/61	55/62	37/62		34/62
			58%		82%	41%	34%	60%	65%	48%	48%	89%	60%		55%
This study; controls	NL	53	6/53		27/53	10/53	5/53	16/53	17/53	5/51	7/53	30/53	10/53		20/51
			11%		51%	19%	9%	30%	32%	10%	13%	57%	19%		39%

A high prevalence of gastro-intestinal symptoms was observed (any GI symptom was mentioned by 82% of the Fabry females, versus 51% in controls). However, none of these individual symptoms were significantly more prevalent in the Fabry disease group with a threshold of p < 0.001 following the Bonferonni correction for multiple comparisons. Nevertheless, five of the nine gastro-intestinal symptoms that were included in the questionnaire were more common in FD females with a p-value of <0.05, with odds ratios ranging from 3 to 5 (Table 2). We feel therefore that it is unlikely that this is completely due to chance and suggests that the gastro-intestinal tract is indeed affected in FD females. In other studies the overall prevalence of gastro-intestinal symptoms in FD females was 34% (range 11-58%). In one large cohort study the prevalence of GI symptoms was 58%, and consisted of complaints of bloating, indigestion and abdominal cramps (Macdermot et al. 2001a, b). A previous smaller study by our group revealed only a prevalence of 12% in the female Fabry patients (Vedder et al. 2007). The differences in prevalence between that study and the current one could be due to difference in methods used (questionnaire versus physicians’ interpretation). The fact that we also observed a high prevalence of gastro-intestinal symptoms in controls (51%) emphasises the need to include a control group, especially when evaluating the prevalence of non-specific symptoms.

This study has identified several potentially FD related symptoms that were previously not recognised. This may be relevant for both FD females and their treating physicians. First, our results indicate that there is a high prevalence of proteinuria during pregnancy in FD females. It cannot be made clear from this study whether these patients had proteinuria before pregnancy, which was subsequently revealed during pregnancy or that Fabry females are more prone to develop proteinuria during pregnancy. However, this finding deserves further study. So far, only case-studies on uneventful pregnancies have been reported (Bouwman et al. 2010; Germain et al. 2010; Kalkum et al. 2009; Parent et al. 2010; Politei 2010; Wendt et al. 2005). The prevalence of hypertension during pregnancy and pre-eclampsia was slightly increased in Fabry females, although not to a statistically significant level in this relatively small group. Its exact pathophysiological mechanism remains unclear. We suggest that complications during pregnancy and pregnancy outcome should be studied in a larger cohort of FD females.

In the current study, we found a high prevalence of loss of libido. This may well be caused by the presence of a chronical medical condition (Simon 2010) and therefore not necessarily be Fabry-specific. Nevertheless it is important for physicians treating FD females to acknowledge presence of this symptom.

A limitation of the current study is that we used a non-validated questionnaire specifically developed for this study. Though a variety of symptoms were evaluated, this questionnaire might not have covered all possible signs and symptoms. Another limitation of the study is the way controls were selected, as this was a patient selected group of friends, neighbours or relatives, and controls were not randomly selected. However, this approach also has advantages, as it generates a control group which is similar in several important respects, such as socioeconomic status and education. In addition, the groups did not differ in age and the controls included in the study were not only healthy individuals. We decided not to stratify for age and treatment with ERT, to prevent additional testing. However, these are important confounders. In general we found that patients treated with ERT had more symptoms and were generally older than patients not on ERT (data not shown). Finally, we confirm that fatigue, palpitations, pains in hands and feet, dizziness and joint pain are more prevalent in FD females as compared to a control group. Although, these symptoms are present in a significant proportion of normal controls they deserve further attention by treating physicians and researchers to better understand their significance, treatment and possible relationship with FD.
